# Complete regression and systemic protective immune responses obtained in B16 melanomas after treatment with LTX-315

**DOI:** 10.1007/s00262-014-1540-0

**Published:** 2014-03-28

**Authors:** Ketil André Camilio, Gerd Berge, Chandra Sekhar Ravuri, Øystein Rekdal, Baldur Sveinbjørnsson

**Affiliations:** 1 Lytix Biopharma AS, P.O. Box 6447, 9294 Tromsø, Norway; 2 Department of Medical Biology, Faculty of Health Sciences, University of Tromsø, MH-Building, Sykehusvegen 44, 9037 Tromsø, Norway; 3 Department of Arctic and Marine Biology, Faculty of Bioscience, Fisheries and Economics, University of Tromsø, Tromsø, Norway; 4 Childhood Cancer Research Unit, Department of Women’s and Children’s Health, Karolinska Institutet, Stockholm, Sweden

**Keywords:** Anticancer peptide, B16 melanoma, Intratumoral treatment, Inflammation, Immunogenic cell death, DAMPs

## Abstract

Malignant melanoma is the most aggressive and deadliest form of skin cancer due to its highly metastatic potential, which calls for new and improved therapies. Cationic antimicrobial peptides (CAPs) are naturally occurring molecules found in most species, in which they play a significant role in the first line of defense against pathogens, and several CAPs have shown promising potential as novel anticancer agents. Structure–activity relationship studies on the CAP bovine lactoferricin allowed us to de novo design short chemically modified lytic anticancer peptides. In the present study, we investigated the in vivo antitumor effects of LTX-315 against intradermally established B16 melanomas in syngeneic mice. Intratumoral administration of LTX-315 resulted in tumor necrosis and the infiltration of immune cells into the tumor parenchyma followed by complete regression of the tumor in the majority of the animals. LTX-315 induced the release of danger-associated molecular pattern molecules such as the high mobility group box-1 protein in vitro and the subsequent upregulation of proinflammatory cytokines such as interleukin (IL) 1β, IL6 and IL18 in vivo. Animals cured by LTX-315 treatment were protected against a re-challenge with live B16 tumor cells both intradermally and intravenously. Together, our data indicate that intratumoral treatment with LTX-315 can provide local tumor control followed by protective immune responses and has potential as a new immunotherapeutic agent.

**Electronic supplementary material:**

The online version of this article (doi:10.1007/s00262-014-1540-0) contains supplementary material, which is available to authorized users.

## Introduction

Malignant melanoma, which develops from a neoplastic transformation of melanocytes, is the most aggressive form of skin cancer, and the number of incidents has increased annually by 2.8 % since 1981 in the US alone [1]. With the increase in the number of patients, the need for new and improved therapies is prevalent. Standard treatment includes surgery, chemotherapy, immunotherapy and radiation therapy [2, 3], while the prognosis and treatment given depends on at which stage the melanoma is diagnosed (Stage I, II, III or IV). Melanomas that have not spread beyond their site of origin are highly curable; however, the difficulties linked to treatment increase considerably once the melanoma metastasizes, thus leading to a poor survival rate [4]. Improvements in treatment regimens have primarily been within immunotherapy, as demonstrated by the approval of Ipilimumab (Yervoy™; Bristol-Myers Squibb, USA) in 2011. Together with ongoing research on targeted therapy, there is an increasing focus on the development of new immunotherapeutic drugs [5, 6].

Cationic antimicrobial peptides (CAPs) are naturally occurring molecules found in a number of species as a defense mechanism for eukaryotic cells against pathogens and are an integral part of the innate immune system [7]. Although there is a vast variation between CAPs when it comes to features such as amino acid sequence and secondary structure, they are normally cationic and amphipathic, which enables them to interact with lipid membranes. CAPs have a broad spectrum of activities such as antimicrobial, anticancer or both, while being less cytotoxic toward non-malignant cells. Nevertheless, some CAPs are non-selective and display cytotoxic activity against microbes and cancer cells, as well as normal mammalian cells [8]. The potential therapeutic application against cancer has spawned an interest in developing anticancer agents that display a new mode of action to overcome the potential drug resistance associated with other current therapeutics. The anticancer effects of CAPs are still under investigation, but several peptides have already exhibited a promising potential with cytotoxic activities against a broad spectrum of tumor cells [9–12]. CAPs with the ability to kill cancer cells are typically referred to as anticancer peptides (ACPs). The anticancer activity of ACPs is exerted through either a membranolytic mode of action or through an interaction with intracellular targets, or a combination of the two. ACPs bind to cancer cell membranes through electrostatic interactions between the positively charged peptide and the anionic cancer cell membrane, thereby leading to a destabilization and disruption of the membrane. The upregulated expression of anionic molecules such as phosphatidylserine [13], sialic acid on glycoproteins [14] and heparan sulfate on proteoglycans [15, 16] on cancer cell membranes results in a higher net negative charge compared to non-malignant eukaryotic cells. This may help explain why ACPs usually have a higher specificity toward certain tumor cells compared to normal eukaryotic cells. Additional factors that may contribute to the selective killing of cancer cells by ACPs include membrane fluidity and cell surface area. Compared to non-malignant cells, cancer cells often have a greater membrane fluidity [17, 18] and cell surface area (additional microvilli) [19, 20], hence leading to an improved anticancer activity of ACPs due to an increased membrane destabilization and the ability to bind more ACP molecules. Moreover, ACPs kill both drug-sensitive and drug-resistant cancer cells due to their membranolytic mode of action, independently of proliferative status and resistance phenotype [21, 22].

Bovine Lactoferricin (LfcinB), which is one of the most widely studied CAPs, has been reported to induce necrosis [23, 24] and apoptosis [25, 26] in vitro, as well as having direct antitumor effect in vivo. Both the systemic administration and intratumoral (i.t.) administration of LfcinB have been shown to inhibit tumor growth and metastasis in several experimental mouse models [23, 24, 27]. Structure–activity relationship (SAR) studies on LfcinB have enabled us to reveal structural parameters critical for antitumor activity, thus leading to the discovery of more potent ACPs [28–32], including the nonapeptide LTX-315. Similar to many ACPs, LTX-315 has the potential to adopt a helical coil structure (predicted by the Garnier-Osguthorpe-Robson V method [33] ). In order to explore the mode of action of LTX-315 in more detail, we investigated the anticancer activities of LTX-315 against a selection of murine and human cancer cell lines in vitro and following i.t. administration against murine melanomas (B16F1) in vivo.

## Materials and methods

### Reagents

LTX-315 (K-K-W-W-K-K-W-Dip-K-NH_2_) and LTX-328 (K-A-Q-Dip-Q-K-Q-A-W-NH_2_) were purchased on request from Bachem AG (Bubendorf, Switzerland) and Innovagen (Lund, Sweden), respectively.

Dacarbazine (D2390), temozolomide (T2577) and *cis*-diammineplatinum dichloride (P4394) were all purchased from Sigma-Aldrich.

### Cell lines

B16F1 (ATCC, CRL-6323), a skin malignant melanoma of C57BL/6 murine origin, MRC-5 (ATCC, CCL-171), a human embryonic lung fibroblast cell line and HUV-EC-C (ATCC, CRL-1730), a human umbilical vein endothelial cell line, were all purchased from the American Type Culture Collection (ATCC-LGC Standards, Rockville, MD, USA). A375 (ECACC, 88113005) is a human malignant melanoma derived from patient material purchased from Public Health England (PHE Culture Collections, Porton Down, Salisbury, UK). B16F1 and A375 cells were cultured in DMEM (high glucose) and MRC-5 cells in MEM (normal glucose) containing 2 mM l-glutamine (all) and 1 % non-essential amino acids (A375 only). Primary epidermal melanocytes (ATCC, PCS-200-013) were cultured in Dermal Basal Medium (ATCC, PCS-200-030) supplemented with the Adult Melanocyte Growth Kit (ATCC, PCS-200-042). HUV-EC-C was cultured using the EGM-2 BulletKit from Lonza, and all growth media were without antibiotics and supplemented with 10 % FBS (except serum-free primary melanocytes). Cell cultures were maintained in a humidified atmosphere of 5 % CO_2_ and >95 % humidity at 37 °C and tested for either both mycoplasma and other pathogens (Rapid-MAPTM-27, Taconic, Europa) or mycoplasma alone.

### In vitro cytotoxicity

A colorimetric 3-(4,5-dimethylthiazol-2-yl)-2,5-diphenyltetrazodium bromide (MTT) viability assay [34] was employed to assess the in vitro cytotoxicity of LTX-315 and LTX-328 (B16F1 only) against a selection of both cancer cells and non-malignant cells. Pre-cultured cells were transferred to a 96-well plate in subconfluent concentrations at a volume of 100 μl/well (culture media). Adherent cells were left overnight in a cell incubator under 37 °C, >95 % humidity and 5 % CO_2_ conditions, and all wells were plated in triplicate. LTX-315 and LTX-328 were dissolved in serum-free RPMI 1640 and diluted to a concentration range of 7–350 μM. Cells were washed once with serum-free RPMI 1640 before being incubated with peptide solutions for 4 h. Cells in serum-free media alone were used as a negative control, while cells treated with 1 % Triton X-100 (Sigma-Aldrich) in a serum-free media as positive controls. A 10 μl MTT solution (5 mg MTT per ml phosphate buffered saline) was added to each well, and the incubation was continued for an additional 2 h. Seventy microliters of solution were removed from each well, and 100 μl of 0.04 M HCl in isopropanol was added before the plates were shaken on an orbital shaker for 1 h at room temperature to facilitate formazan crystal solubilization. The absorbance was measured at 590 nm on a microtiter plate reader (Thermomax Molecular Devices, NJ, USA). The percentage of viability was calculated as the A_590_ nm of peptide-treated cells relative to the negative control (100 % living cells) and expressed as a 50 % inhibitory concentration (IC_50_).

### LTX-315 kinetics study

The kill kinetics of LTX-315 against B16F1 melanoma cells was studied using the IC_100_ concentration of LTX-315 (35 μM, after 1 h). Cells were washed once with serum-free RPMI 1640 before being incubated with peptide solution for 5, 15, 30, 45, 60, 90 and 120 min. After incubation with peptide, the cells were washed once and further incubated for 2 h with serum-free RPMI 1640 containing 10 % MTT.

### LTX-315 versus cytostatica

LTX-315 and the different cytostatic drugs were dissolved in serum-free RPMI 1640 to a concentration range of 7–100 μM (LTX-315) and 10–100 μM (cytostatic drugs), respectively. The cytostatic drugs were originally dissolved in dimethyl sulfoxide (DMSO) and later in serum-free RPMI 1640, yielding a final DMSO concentration ranging from 0.5 % in the 100 μM solution to 0.05 % in the 10 μM solution. Cells were washed once with serum-free RPMI 1640 before being incubated with the peptide solutions or cytostatic drugs for 2, 4, 8, 24 and 48 h, respectively.

### Hemolytic activity

Freshly isolated human red blood cells (hRBCs), prepared as previously described [23], were incubated for 1 h at 37 °C with LTX-315 dissolved in PBS at concentrations ranging from 0.7 to 695 μM. The samples were centrifuged at 2,000 g for 5 min before the absorbance of the supernatant (S) was measured at 405 nm by a spectrophotometric microtiter plate reader (Thermomax Molecular Devices, NJ, USA). The 0 and 100 % hemolysis controls consisted of hRBCs treated with PBS and 0.1 % Triton X-100 in PBS, respectively.

### Release of high mobility group box-1 protein (HMGB1) from B16F1 cells

Murine B16F1 cells (3 × 10^5^/well) were seeded in a 6-well plate, treated with either 35 μM LTX-315 (IC_100_) or 35 μM LTX-328 and incubated at 37 °C at 5 % CO_2_ and >95 % humidity for different time points (5–60 min). Serum-free RPMI 1640 was used as a negative control. Supernatants were collected after centrifugation at 1,400 g for 5 min and up-concentrated using Amicon Ultra-0.5 Centrifugal Filter units with Ultracel-50 membrane (Millipore, Norway). Cell lysates were washed with serum-free RPMI-1640 and collected in a Mastermix containing a 2× NuPAGE LDS Sample buffer (Invitrogen, Norway), a 1× NuPAGE Sample Reducing Agent (Invitrogen, Norway) and 50 % sterile H_2_O. Supernatants and cell lysates were boiled in a reducing NuPAGE LDS sample buffer, resolved on NuPAGE Novex 4–12 % Bis–Tris Gels and electrotransferred onto a polyvinylidene difluoride (PVDF) membrane (Millipore, Norway). Membranes were hybridized with HMGB1 antibody (rabbit polyclonal to HMGB1—ChIP Grade, Abcam, UK), followed by horseradish peroxidase (HRP)-conjugated secondary antibody (goat polyclonal anti-rabbit IgG, Abcam, UK) and developed by Western blot luminol reagent (Santa Cruz Biotechnology, USA) according to the manufacturer’s instructions.

### Animals

Female C57BL/6 wild-type mice, 5–6 weeks old, were obtained from Charles River, United Kingdom. All mice were housed in cages in a pathogen-free animal facility according to local and European Ethical Committee guidelines.

### Tumor treatment

Tumor cells were harvested, washed in RPMI-1640 and injected intradermally (i.d.) into the right side of the abdomen in C57BL/6 mice (5 × 10^4^ B16F1 cells per mouse/50 μl RPMI-1640). Palpable tumors (20–30 mm^2^) were injected i.t. with single doses of LTX-315 or LTX-328 dissolved in saline (1.0 mg peptide/50 μl saline) once a day for 3 consecutive days, and the vehicle control was saline only (0.9 % NaCl in sterile H_2_O). Tumor size was measured using an electronic caliper and expressed as the area of an ellipse [(maximum dimension/2) × (minimum dimension/2) × π]. Animals were then euthanized when the product of the perpendicular tumor dimensions reached 130 mm^2^ or when tumor ulceration developed.

### Histological examination

Tissues were fixated by perfusion fixation using 4 % paraformaldehyde. Following fixation and paraffin embedding, tissue sections were deparaffinized in xylene and graded alcohols, before being hydrated and washed with PBS. After antigen retrieval in a sodium citrate buffer (pH 6) in a microwave oven, the endogenous peroxidase was blocked by 0.3 % H_2_O_2_ for 15 min. Sections were then incubated overnight at 4 °C with a primary antibody (rabbit anti-CD3; Dako). As a secondary antibody, an anti-rabbit-HRP SuperPicTure Polymer detection kit was used (Invitrogen, San Francisco, CA, USA). A matched isotype control was further used as a control for non-specific background staining. A routine standard staining was performed with hematoxylin and eosin.

### mRNA analysis using reverse transcriptase quantitative PCR

B16 melanoma tumor tissue was harvested at selected time points (4, 24, 48 and 120 h) following a single i.t. injection of either LTX-315 (1 mg) or the vehicle control. Animals were euthanized using CO_2_ gas, and tumor tissue was harvested and put on Allprotect Tissue Reagent (Qiagen, Hilden, Germany) and left overnight at 4 °C. Samples were later stored at −20 °C until use.

Total RNA was extracted from the tumors using the RNeasy Plus Universal Mini kit (Qiagen, Hilden, Germany), and on-column DNase treatment of the RNA samples was performed using RNase-Free DNase (Qiagen, Hilden, Germany) according to the manufacturer’s instructions. The quantity and purity of the extracted RNA were determined using the NanoDrop spectrophotometer (Thermo Fisher Scientific, Wilmington, DE, USA), while the integrity of RNA was checked by the Experion automated electrophoresis system from Bio-Rad.

The mRNA expression levels in the tumors were quantified by reverse transcription quantitative PCR (RT-qPCR) performed on a Stratagene Mx3000P instrument (Stratagene, La Jolla, CA, USA). A reverse transcription of total RNA was performed using the QuantiTect Reverse Transcription Kit (Qiagen, Hilden, Germany) with 1 μg of RNA per 20 μl cDNA reaction. cDNA corresponding to 10 ng RNA was amplified for 40 cycles in a 25-μl PCR mix (Brilliant II SYBR^®^ Green Low ROX QPCR Master Mix, Agilent Technologies, Santa Clara, CA, USA) containing a final concentration of 240 nM (for IL1 β, IL6, IL18, TNF-α, GAPDH and ACT-B) and 400 nM (for IL2 and IFN-γ) of each primer (Supplementary Table 1). Cycling conditions were as follows: 95 °C for 10 min, 40 cycles at 95 °C for 30 s, 55 °C for 1 min, 72 °C for 30 s and 1 cycle at 95 °C for 1 min. Primer specificities and an absence of primer dimers were determined by the use of a SYBR green melting curve analysis. Duplicate reverse transcriptase reactions were performed for each RNA sample, and a duplicate PCR analysis was performed for each cDNA sample. The absence of genomic DNA was confirmed by performing a no reverse transcriptase (NoRT) control for randomly selected RNA samples, and the absence of contaminations was assessed by including a no template control (NTC) in every run. The ΔΔ*C*_T_ method [35] was used to determine the relative amount of target genes, normalizing against the average expression of the two reference genes, GAPDH and ACT-B.

### ELISA analysis of plasma

Blood from C57BL/6 mice with palpable tumors was harvested at selected time points (4, 24, 48 and 120 h) following a single i.t. injection of either LTX-315 (1 mg) or the vehicle control. The blood was transferred to a MiniCollect EDTA-K2 (Fisher Scientific) 0.5-ml tube and kept on ice before being centrifuged at 1,000 g for 10 min. Plasma was analyzed for IL1 β/IL1 F2 and IL6 using the Quantikine^®^ ELISA from R&D Systems.

### Secondary tumor challenge

Animals with a complete regression (CR) of tumor after LTX-315 treatment were given a second i.d. tumor cell challenge (5 × 10^4^ B16F1 cells) on the left-hand side of the abdomen (contralateral to the first tumor site) 4–5 weeks after they were cured by LTX-315. Animals surviving the i.d. tumor re-challenge were later given a second tumor re-challenge intravenously (i.v.) (2 × 10^5^ cells) through the tail vein. Lungs were harvested on day 19 after the i.v. re-challenge. All mice were monitored for tumor size and survival.

### Statistical analysis

All data represent at least three independent experiments and are expressed as the mean ± the standard deviation (SD) or the standard error of mean (SEM). Animal survival curves (Kaplan–Meier Plot) were compared using a log-rank (Mantel-Cox) test. Real-time PCR data were compared using one-way ANOVA and a Tukey’s multiple comparison test, and we considered *p* values ≤0.05 to indicate statistical significance.

## Results

### Malignant melanoma cells are more sensitive to LTX-315 compared to non-malignant cells

**Fig. 1:**
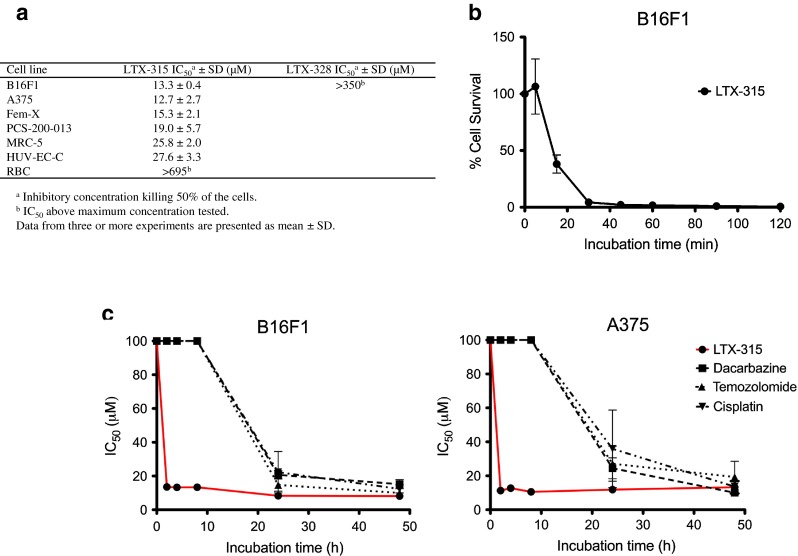
In vitro cytotoxicity of LTX-315, B16 melanoma cell cytocide kinetics and in vitro cytotoxicity of LTX-315 versus chemotherapeutic drugs. The in vitro cytotoxicity of LTX-315 and LTX-328 against a selection of cancer cell lines and normal cell lines (**a**). Cell killing kinetics of LTX-315 (IC_100_) against B16F1 melanoma cells (**b**) after designated time points (5, 15, 30, 45, 60, 90 and 120 min). IC_100_ of LTX-315 = 35 μM. In vitro cytotoxicity data demonstrating IC_50_ values of LTX-315 (*red line*) and three different chemotherapeutic drugs (**c**), dacarbazine (*filled squares*), temozolomide (*filled triangles*) and cisplatin (*filled reverse triangles*) after designated time points (2, 4, 8, 24 and 48 h). The four compounds were tested against a murine B16F1 and a human A375 melanoma cell line. Data from three experiments are presented for each time point as mean ± SEM

The cytotoxicity of LTX-315 was examined against a panel of murine and human melanoma cell lines and untransformed cell lines using the MTT assay [34]. LTX-315 exhibited a twofold higher activity toward the three melanoma cell lines, B16F1 (murine), Fem-X and A375 (human) compared to normal human fibroblasts (MRC-5) and endothelial cells (HUV-EC-C). The peptide exhibited a slightly higher cytotoxicity toward human melanoma cells (A375) than normal human primary melanocytes (PCS-200-013). There was no detectable IC_50_ hemolytic activity by LTX-315 within the concentration range tested. The nonapeptide LTX-328 is a non-lytic control peptide that was used as a control peptide to LTX-315. No cytotoxic activity was observed for LTX-328 against B16F1 melanoma cells (Fig. 1a).

### LTX-315 rapidly kills B16 melanoma cells in vitro

The kill kinetics of LTX-315 against B16F1 melanoma cells was studied by treating B16F1 cells with LTX-315 for different time points. Figure 1b shows that the peptide lyses B16F1 cells within 15 min of treatment. At 30 min post-treatment, nearly all cells were dead, thereby demonstrating a rapid lytic mode of action for LTX-315 against B16 melanoma cells.

### LTX-315 displays rapid kill kinetics compared to conventional chemotherapeutics

The cytotoxic effect of LTX-315 was compared with commonly used chemotherapeutic drugs against melanoma (dacarbazine, temozolomide and cisplatin), using the MTT assay. LTX-315 exhibited a more rapid cytotoxic effect against both murine and human melanoma cells (B16F1 and A375, respectively) compared to the chemotherapeutic drugs, as shown by an IC_50_ value of 13 μM (B16F1) and 11 μM (A375) 2 h post-peptide treatment, compared to >100 μM for all the chemotherapeutics. A significant anticancer effect was first observed after 24 h for the chemotherapeutic drugs (Fig. 1c).

### B16F1 melanoma cells treated with LTX-315 in vitro release high mobility group box-1 (HMGB1)

**Fig. 2:**
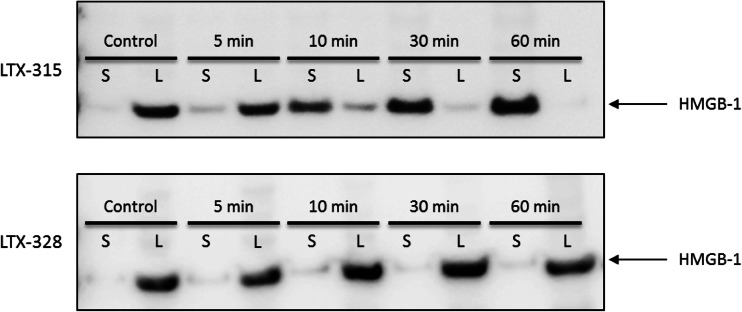
LTX-315 treatment leads to extracellular release of HMGB1 protein. B16F1 melanoma cells were treated in vitro with 35 μM of either LTX-315 (*top*) or LTX-328 (*bottom*) for selected time points (5–60 min). The translocation of the HMGB1 protein from the lysate (L) to the supernatant (S) was shown using Western blot. Control cells showed no translocation after 60 min. The figure is a digital image of a representative blot of three experiments

To study whether LTX-315 was able to induce the release of danger-associated molecular pattern molecules (DAMPs), which is one of the requirements for immunogenic cell death, the release of HMGB1 from LTX-315- and LTX-328-treated B16F1 melanoma cells was measured using Western blot. B16F1 cells were treated with 35 μM of either LTX-315 or LTX-328, and the translocation of HMGB1 (normally expressed within the nucleus) from the lysate (L) to the supernatant (S) was assessed. Melanoma cells treated with LTX-315 showed a gradual translocation of HMGB1 from the lysate to the supernatant after peptide treatment, with an increasing concentration in the supernatant as time progressed. After 30 min of treatment with LTX-315, most of the HMGB1 was released into the supernatant. Untreated control cells and cells treated with LTX-328 showed no translocation of HMGB1, and the protein was retained within the lysates during the entire incubation period (Fig. 2).

### LTX-315 induces complete regression of palpable B16F1 tumors following intratumoral injection

**Fig. 3:**
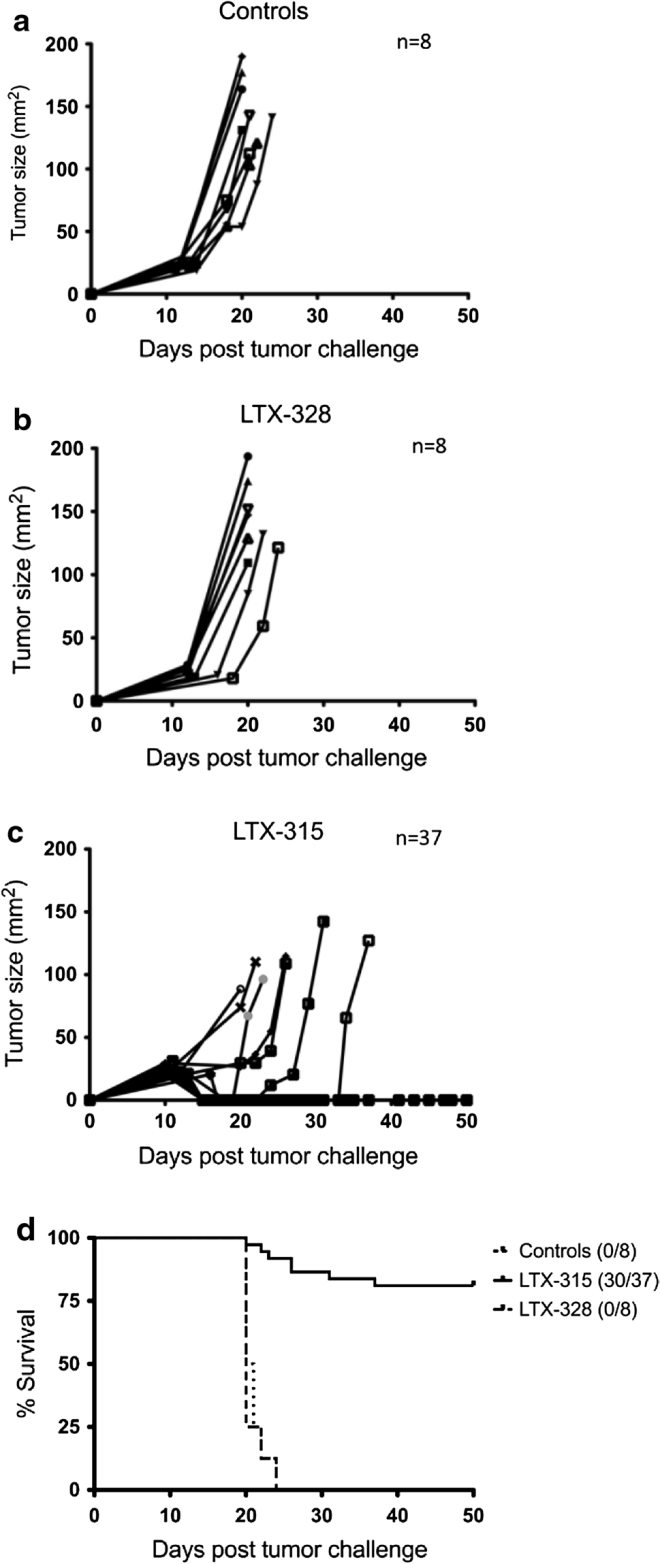
Effect of LTX-315 treatment on tumor growth of murine B16 melanomas. Palpable melanoma tumors syngeneic with C57BL/6 mice were injected intratumorally with sterile 0.9 % NaCl (vehicle controls) (**a**), with 1 mg LTX-328 (**b**), or with 1 mg LTX-315 (**c**) once per day on day 12, 13 and 14 after tumor challenge. The survival curves (**d**) were analyzed using a log-rank (Mantel-Cox) test and were shown to be significantly different (*p* = 0.0005)

To investigate the therapeutic potential of LTX-315, C57BL/6 mice with B16 melanomas were injected i.t. with 50 μl of either vehicle (*n* = 8), 20 mg/ml LTX-315 (*n* = 37) or LTX-328 (*n* = 8) for three consecutive days. In the control group and LTX-328 group, tumors developed rapidly and animals had to be euthanized due to the tumor burden (130 mm^2^) within 25 days after tumor challenge. Tumor growth was not affected by injections with either the vehicle or LTX-328. In the LTX-315-treated group, complete regression (CR) of tumor tissue was observed in most animals (Fig. 3). The median survival was >50 days for LTX-315-treated animals, compared to 20 and 20.5 days for saline and LTX-328-injected animals, respectively. At the end of study (50 days post-tumor challenge), 30 out of the 37 animals treated with LTX-315 had CR. Treatment with LTX-315 induced rapid tumor cell lysis, which was seen as a visible necrotic tumor area 24 h after the first i.t. injection (data not shown).

### Intratumoral treatment with LTX-315 stimulates T cell infiltration

**Fig. 4:**
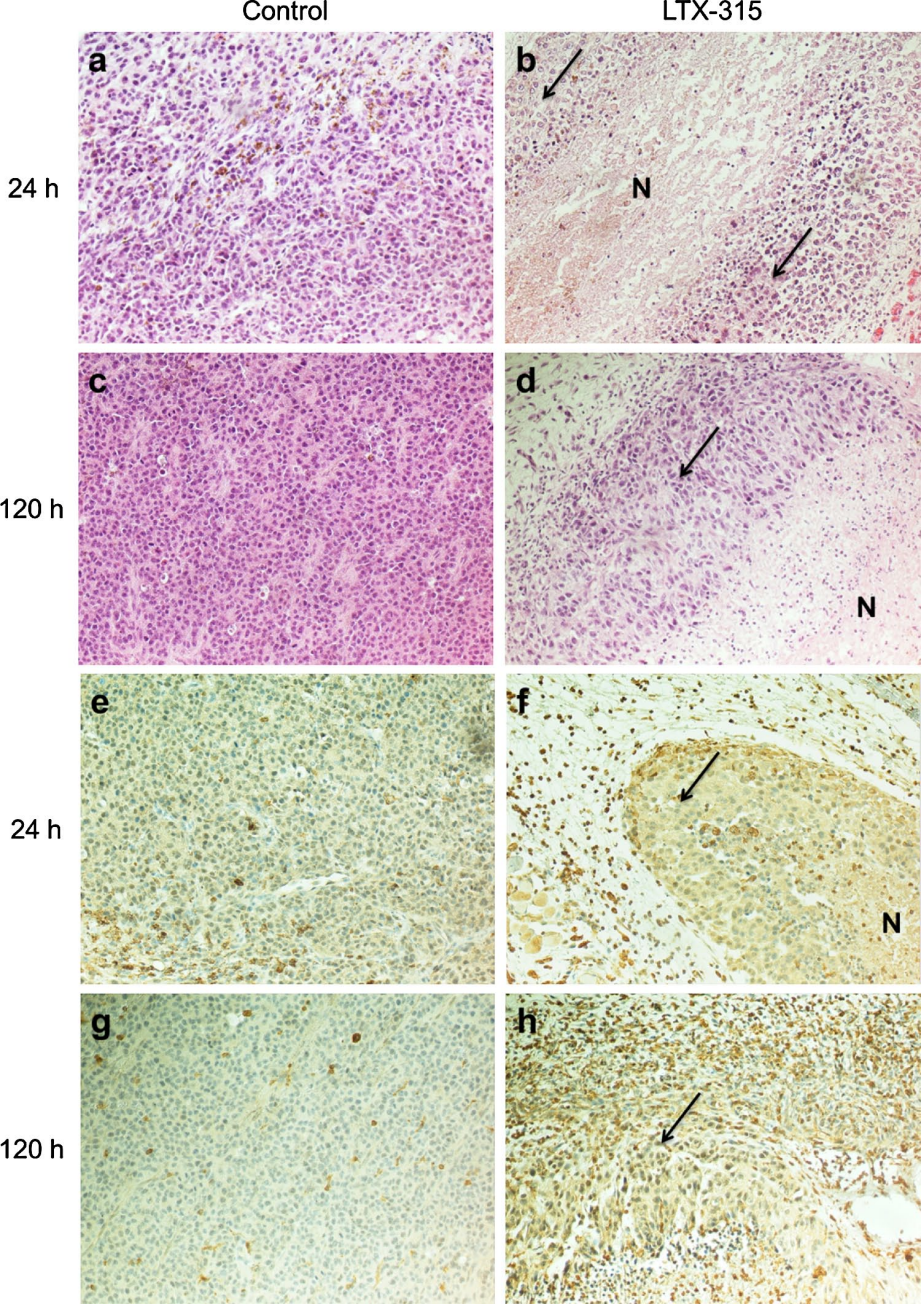
Intratumoral treatment with LTX-315 induces immune cell infiltration. B16 tumors were surgically excised 24 h and 120 h post-injection with vehicle (**a**, **c**, **e** and **g**) or LTX-315 (**b**, **d**, **f** and **h**). Tumors injected with LTX-315 exhibited tumor tissue necrosis (**N**) and immune cell infiltration 24 h post-injection (**b**) with an increased cell infiltration 120 h post-treatment (**d**), compared to viable controls (**a** and **c**). Immunolabeling with anti-CD3 showed many of the infiltrating cells to be CD3^+^ T cells (**f** and **h**), compared to low- or non-infiltrated control tumors (**e** and **g**). The *arrows* point to the remaining viable tumor tissue in treated tumors. Histological examinations were performed by H&E staining and anti-CD3 immunolabeling at ×200

In order to assess histological changes following local injection with LTX-315, tumors were perfusion fixated and surgically excised from animals (*n* = 2) after a single i.t. injection of vehicle (controls) or 1 mg LTX-315 at selected time points (4, 24, 48 and 120 h). A histological examination of the tumors revealed that i.t. injection of LTX-315 induced an extensive necrosis of the tumor tissue, followed by a massive infiltration of lymphocytes into both the surrounding connective tissue and the tumor parenchyma (Fig. 4b, d). Immunolabeling with anti-CD3 showed that many of the infiltrating cells were CD3^+^ T cells (Fig. 4f, h), whereas tumors injected with vehicle exhibited a viable tissue with no necrosis and a low number of lymphocytes (Fig. 4a, c, e, g).

### Intratumoral treatment with LTX-315 mounts an inflammatory response in B16 tumors

**Fig. 5:**
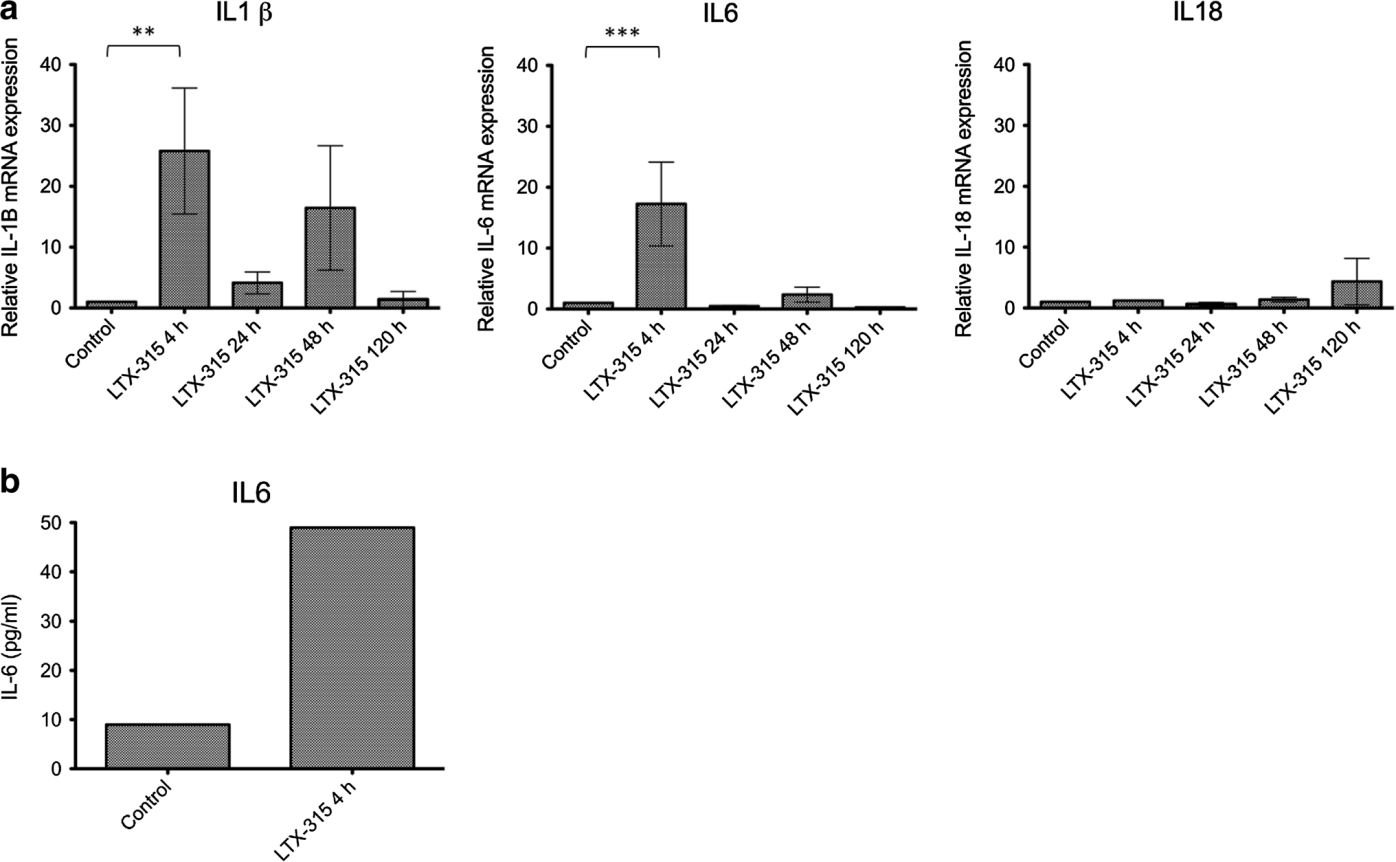
Intratumoral treatment with LTX-315 induces an inflammatory response. Following a single i.t. injection of sterile 0.9 % NaCl (control), or with 1 mg LTX-315, tumor tissue (**a**) and plasma (**b**) were harvested 4, 24, 48 and 120 h post-injection and analyzed for mRNA expression of several different cytokines using RT qPCR (tumor tissue) or for cytokine content using ELISA (plasma). Data from three animals are presented for each time point as mean ± SEM. Normal tumor expression (control) was defined as 1 (normal expression = normal/normal), while treated tumor expression was defined as treated tumor = treated tumor/normal (**a**). Statistical analysis was performed using a Tukey′s multiple comparison test (*p* < 0.05). Data from animals treated with either LTX-315 (*n* = 4) and vehicle (*n* = 2) are presented as the pg/ml content of IL6 in pooled plasma samples (**b**)

To elucidate the mechanism behind the inflammatory response induced by LTX-315 in vivo and investigate the potential immune-modulating properties of the peptide, B16 melanomas treated with a single i.t. injection of vehicle (controls) or 1 mg of LTX-315 (peptide treated) were harvested from animals (*n* = 3) at selected time points (4, 24, 48 and 120 h). Tumor tissue was analyzed for relative mRNA expression of a panel of different cytokines (IL1 β, IL2, IL6, IL18, TNF-α and IFN-γ, Supplementary Table 1). As early as after 4 h, an increased expression of the pro-inflammatory cytokines IL1 β and IL6 was evident, which was significantly upregulated compared to the controls injected with the vehicle. A certain increment in the expression of IL1 β was also seen after 48 h. Moreover, IL18 exhibited an increased mRNA expression in LTX-315-treated tumors 120 h post-injection (Fig. 5a). LTX-315-treated tumors did not show an increased mRNA expression of IL2, TNF-α and IFN-γ compared to vehicle-injected control tumors (data not shown).

### ELISA analysis of plasma

To analyze for pro-inflammatory cytokines in animals injected with either a single injection of LTX-315 (*n* = 4) or the vehicle control, (*n* = 2) plasma was harvested from animals at selected time points (4, 24, 48 and 120 h). The plasma was analyzed for IL1 β and IL6. An increased level of IL6 was observed in the plasma 4 h after treatment with LTX-315 compared to vehicle controls (Fig. 5b). LTX-315-treated animals did not show an increased level of IL1 β in the plasma compared to vehicle-injected control tumors (data not shown).

### LTX-315 treatment of B16 melanomas induces protective immune responses

**Fig. 6:**
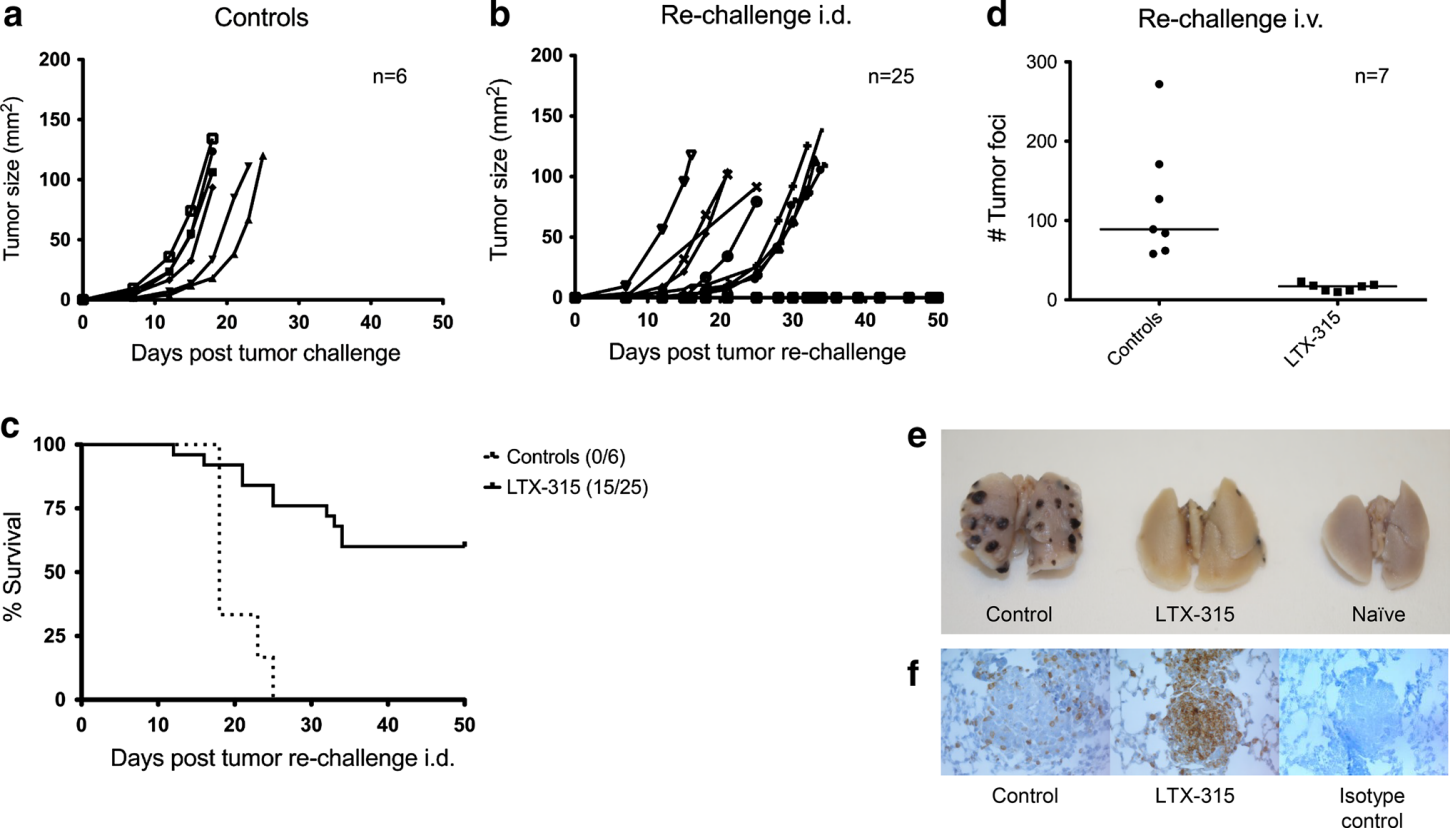
LTX-315 treatment induces immune protection against B16 melanomas. Tumor growth in non-treated control animals (**a**) was compared to animals previously cured by LTX-315 treatment (**b** and **d**). Animals were re-challenged intradermally with 5 × 10^4^ viable B16F1 cells contralateral to the first tumor site (**b**) or intravenously with 2 × 10^5^ viable B16F1 cells (**d**). The survival curves of animals re-challenged intradermally **(c)** were analyzed using a log-rank (Mantel-Cox) test and were shown to be significantly different (*p* < 0.0001). The number of lung tumor foci was analyzed using an unpaired *t* test and was shown to be significantly different (*p* = 0.003) when comparing intravenously re-challenged animals previously cured by LTX-315 with non-treated control animals (**d**). A digital image illustrates representative lungs from the different groups (**e**). The tumor foci of animals previously cured by LTX-315 were highly infiltrated by CD3^+^ T cells compared to control animals as shown by immunolabeling with anti-CD3 (**f**)

To examine whether treatment with LTX-315 was able to induce adaptive immune responses, cured animals (*n* = 25), together with non-treated control animals (*n* = 6), were re-challenged with 5 × 10^4^ viable tumor cells i.d. on the abdomen contralateral to the first tumor site 4–5 weeks after CR was attained. Animals achieving CR following the i.d. tumor re-challenge were later re-challenged a second time i.v. The majority of animals cured by LTX-315 showed growth inhibition, and tumor growth was absent in 15 out of the 25 animals, while all the control animals developed tumors subsequent of i.d. re-challenge (Fig. 6c). Animals previously cured by LTX-315 i.t. injections (*n* = 7) showed systemic immune protection against B16 melanomas following i.v. re-challenge (Fig. 6d, e), compared to non-treated control animals (*n* = 7). LTX-315-cured animals (mean # tumor foci = 15.86) displayed a significantly lower number of lung tumor foci compared to non-treated control animals (mean # tumor foci = 123.3). Additionally, histological examinations revealed that the lung tumor foci of animals previously cured by LTX-315 were significantly more infiltrated by CD3^+^ T cells, compared to the less infiltrated lung tumor foci of non-treated control animals (Fig. 6f).

## Discussion

Cationic antimicrobial peptides have been studied since the 1960s [36, 37] and in later years have received added attention for their therapeutic potential against cancer [9–11]. Our group has previously shown that CAPs such as LfcinB can induce complete tumor regression following the i.t. treatment of syngeneic tumors in mouse models [23] and tumor growth inhibition in a human xenograft model [24]. Through SAR studies on LfcinB derivatives, we were able to synthesize shorter and more effective CAPs with an improved anticancer activity, both in vitro and in vivo [28–32]. LTX-315 was designed as a helical CAP optimized for membrane destabilization to be used for the local treatment of solid tumors.

Previous studies have shown that naturally occurring CAPs such as the cell-penetrating peptide, crotamine, display potent cytotoxic activity against B16 melanoma cells in vitro as well as B16 melanomas established in syngeneic mice [38]. In the present study, the antitumor effect of LTX-315 against the highly aggressive and low immunogenic B16 melanoma cell line [39] was studied. In vitro results revealed that LTX-315 was highly active against both murine and human melanoma cell lines. All the cancer cell lines were more sensitive to LTX-315 compared to normal human fibroblasts (MRC-5) and endothelial cells (HUV-EC-C). In accordance with studies on other CAPs, the specificity of LTX-315 for tumor cells is probably due to differences in cell membrane composition between cancer cells and non-malignant cells. Cancer cells have been shown to have a larger quantity of anionic molecules on their membrane [13, 14, 16], in addition to an increased membrane fluidity [17, 18] and membrane cell surface due to more microvilli [19, 20]. The cytotoxicity of LTX-315 was somewhat similar toward human melanoma cells (A375) and normal human primary melanocytes (PCS-200-013). However, since LTX-315 is designed for intratumoral treatment, the selectivity of the peptide is of less significance compared to drugs used systemically. Locally delivered cytotoxic drugs have been shown to increase cancer cell killing and improve clinical responses, in addition to minimize patient toxicity, compared to systemically delivered drugs [40]. LTX-315 did not display any IC_50_ cytotoxic activity against hRBCs (Fig. 1a). Red blood cell membranes are neutral due to their high content of neutral phospholipids such as sphingomyelin and phosphatidylcholine [41], demonstrating that LTX-315 is membrane-specific. LTX-315 induced the rapid killing of B16F1 and A375 cells compared to conventional chemotherapeutics (Fig. 1b, c), which kill cells by interfering with DNA replication or structure [42–44].

B16F1 cells treated with LTX-315 in vitro released HMGB1 from the inside of the cells to the cell supernatant (Fig. 2), where it can act as a DAMP. DAMPs are able to stimulate the maturation of dendritic cells (DCs), thereby leading to an enhancement of responses from CD8^+^ T cells [45, 46]. Normally in the nuclear compartment, when released into the extracellular environment, HMGB1 has been reported to be involved in the recruitment of inflammatory cells [47] and the promotion of natural killer (NK) cells, including monocyte interactions, DC maturation [48] and the increase of IFN-γ release by NK cells [49]. By inducing necrotic cell death, LTX-315 is capable of stimulating the immune system due to the release of DAMPs, thus leading to an inflammatory reaction. A failure to induce the release of DAMPs will not stimulate the immune system, and potential cellular death will therefore be non-immunogenic [46].

The anticancer effect of LTX-315 was examined in a mouse melanoma model through the i.t. injection of intradermal B16 tumors. A majority of the animals treated with LTX-315 experienced a complete and long-lasting tumor regression and were tumor-free 5 weeks post-treatment (Fig. 3). A histological examination revealed that LTX-315 induced extensive hemorrhagic necrosis of the tumor parenchyma and massive infiltration of lymphocytes. Immunolabeling with anti-CD3 showed that a major portion of the infiltrating immune cells were T cells (Fig. 4), indicating that LTX-315 induces a cell death that leads to an increase in the number of tumor-infiltrating lymphocytes (TILs).

The presence of an inflammatory response in the B16 melanomas treated with LTX-315 was further indicated by increased mRNA levels of inflammatory cytokines such as interleukin (IL) 1β, IL6 and IL18 in the tumor tissue and of IL6 in plasma samples. Expression of IL1 β and IL6 was augmented in LTX-315-treated tumors compared to vehicle-injected tumors (*p* < 0.05), and there were also indications of an increased expression of IL18 in peptide-treated tumors compared to control tumors (not significant) (Fig. 5a). There was a fivefold increase of IL6 in the plasma of animals injected with LTX-315 compared to vehicle-injected control animals 4 h post-injection. IL1 β has been shown to promote the infiltration of inflammatory and immunocompetent cells, function as a co-stimulator of T cell functions (usually together with an antigen or a mitogen) and play a role in antibody production from B cells [50, 51]. Interleukin 6 is involved in acute inflammation following tissue trauma or damage, in T_H_17 cell induction and differentiation and in B cell proliferation and antibody production [50, 52, 53]. Interleukin 18 is involved in both innate and adaptive immune responses such as inflammation, modulates the activity of T_H_1 cells, inhibits angiogenesis and has chemoattractant properties for polymorphonuclear cells [50, 54–57]. The upregulation of these cytokines demonstrates that local treatment of B16 melanomas with LTX-315 stimulates a pro-inflammatory response and an activation of the immune system. We did not detect increased mRNA levels for TNF-α and IFN-γ in LTX-315-treated tumors compared to vehicle-injected tumors. Cytokine gene expression is tightly regulated, and the mRNAs for most cytokines have been shown to have a very short half-life [58]. TNF-α mRNA is normally degraded within minutes and therefore often difficult to detect.

To investigate whether the immune-modulating properties of LTX-315 in vivo led to a long-term protective immune response, animals were re-challenged with live tumor cells. All previously cured animals demonstrated significant tumor growth inhibition and no tumor take was observed in 60 % of the animals given viable cells i.d. (Fig. 6a–c). Animals surviving the i.d. re-challenge were re-challenged with viable B16 melanoma cells i.v. 9 months post-LTX-315 treatment to examine a possible long-lasting systemic protection against the cancer and its effect in an experimental lung metastasis model. All animals re-challenged i.v. demonstrated systemic immune protection against B16 melanomas compared to non-treated control animals (Fig. 6d, e) in addition to an increase in the amount of infiltrating CD3^+^ T cells into the re-challenged lung tumor foci, as seen by immunolabeling with anti-CD3 (Fig. 6f). This indicates that LTX-315 induced systemic antitumor immune responses, with the persistence of a T cell-mediated antitumor immunity and thus a long-term protection against relapse of the tumor and against lung tumor foci. Berge et al. [28] have previously shown that the immune protection is transferrable between animals using adoptive transfer techniques, after i.t. treatment of syngeneic A20 lymphomas with a peptide analog to LTX-315, named LTX-302, demonstrating that the immune protection is T cell dependent. The mechanism of LTX-315 is thought to be similar to that of LTX-302, by inducing long-term, specific cellular immunity against B16 melanomas through membrane-induced lysis and the extracellular release of DAMPs (HMGB1). This LTX-315-induced immunogenic cell death potentially results in the maturation of antigen-presenting cells such as DCs and a subsequent presentation of tumor antigens to T cells, creating specific cytotoxic T cells capable of eradicating residual cancer cells.

Taken together, our observations in vitro and in vivo indicate that i.t. administration of LTX-315 leads to extensive tumor necrosis initiated by a direct disruptive effect of the peptide on the plasma membrane of tumor cells. Moreover, the necrotic effect of LTX-315 leads to the release of DAMPs that stimulates immune responses and the infiltration of TILs into the tumor parenchyma, which may be critical in the eradication of solid B16 melanomas due to their possible role in inducing a long-lasting tumor immune protection.

## Electronic supplementary material

Below is the link to the electronic supplementary material.

Supplementary material 1 (PDF 91 kb)

## Abbreviations

ACPs: Anticancer peptides
CAPs: Cationic antimicrobial peptides
DAMPs: Danger-associated molecular pattern molecules
DCs: Dendritic cells
HMGB1: High mobility group box-1
hRBCs: Human red blood cells
i.d.: Intradermal
IL: Interleukin
i.t.: Intratumoral
LfcinB: Bovine lactoferricin
MTT: 3-(4,5-dimethylthiazol-2-yl)-2,5-diphenyltetrazodium bromide
NK: Natural killer cells
SAR: Structure–activity relationship
TILs: Tumor-infiltrating lymphocytes

## References

[1] Little EG, Eide MJ (2012). Update on the current state of melanoma incidence. Dermatol Clin.

[2] Duran Garcia E, Santolaya R, Requena T (1999). Treatment of malignant melanoma. Ann Pharmacother.

[3] McNeer G, Das Gupta T (1965). Treatment of malignant melanoma. CA Cancer J Clin.

[4] Balch CM, Buzaid AC, Soong SJ, Atkins MB, Cascinelli N (2001). Final version of the American Joint Committee on Cancer staging system for cutaneous melanoma. J Clin Oncol.

[5] Eggermont AM, Robert C (2011). New drugs in melanoma: it’s a whole new world. Eur J Cancer.

[6] Spagnolo F, Queirolo P (2012). Upcoming strategies for the treatment of metastatic melanoma. Arch Dermatol Res.

[7] Zasloff M (2002). Antimicrobial peptides of multicellular organisms. Nature.

[8] Pasupuleti M, Schmidtchen A, Malmsten M (2012). Antimicrobial peptides: key components of the innate immune system. Crit Rev Biotechnol.

[9] Hoskin DW, Ramamoorthy A (2008). Studies on anticancer activities of antimicrobial peptides. Biochim Biophys Acta.

[10] Mader JS, Hoskin DW (2006). Cationic antimicrobial peptides as novel cytotoxic agents for cancer treatment. Expert Opin Investig Drugs.

[11] Schweizer F (2009). Cationic amphiphilic peptides with cancer-selective toxicity. Eur J Pharmacol.

[12] Riedl S, Zweytick D, Lohner K (2011). Membrane-active host defense peptides—challenges and perspectives for the development of novel anticancer drugs. Chem Phys Lipids.

[13] Utsugi T, Schroit AJ, Connor J, Bucana CD, Fidler IJ (1991). Elevated expression of phosphatidylserine in the outer membrane leaflet of human tumor cells and recognition by activated human blood monocytes. Cancer Res.

[14] Marth E, Flaschka G, Stiegler S, Mose JR (1988). Sialic-acid as a marker for differentiation between benign and malignant intracranial tumors. Clin Chim Acta.

[15] Fadnes B, Uhlin-Hansen L, Lindin I, Rekdal O (2011). Small lytic peptides escape the inhibitory effect of heparan sulfate on the surface of cancer cells. BMC Cancer.

[16] Kleeff J, Ishiwata T, Kumbasar A, Friess H, Buchler MW (1998). The cell-surface heparan sulfate proteoglycan glypican-1 regulates growth factor action in pancreatic carcinoma cells and is overexpressed in human pancreatic cancer. J Clin Invest.

[17] Kozłowska K, Nowak J, Kwiatkowski B, Cichorek M (1999). ESR study of plasmatic membrane of the transplantable melanoma cells in relation to their biological properties. Exp Toxicol Pathol.

[18] Sok M, Sentjurc M, Schara M (1999). Membrane fluidity characteristics of human lung cancer. Cancer Lett.

[19] Chaudhary J, Munshi M (1995). Scanning electron microscopic analysis of breast aspirates. Cytopathology.

[20] Domagala W, Koss L (1980). Surface configuration of human tumor cells obtained by fine needle aspiration biopsy. Scan Electron Microsc.

[21] Johnstone SA, Gelmon K, Mayer LD, Hancock RE, Bally MB (2000). In vitro characterization of the anticancer activity of membrane-active cationic peptides. I. Peptide-mediated cytotoxicity and peptide-enhanced cytotoxic activity of doxorubicin against wild-type and p-glycoprotein over-expressing tumor cell lines. Anticancer Drug Des.

[22] Kim S, Kim SS, Bang YJ, Kim SJ, Lee BJ (2003). In vitro activities of native and designed peptide antibiotics against drug sensitive and resistant tumor cell lines. Peptides.

[23] Eliassen LT, Berge G, Sveinbjornsson B, Svendsen JS, Vorland LH (2002). Evidence for a direct antitumor mechanism of action of bovine lactoferricin. Anticancer Res.

[24] Eliassen LT, Berge G, Leknessund A, Wikman M, Lindin I (2006). The antimicrobial peptide, lactoferricin B, is cytotoxic to neuroblastoma cells in vitro and inhibits xenograft growth in vivo. Int J Cancer.

[25] Mader JS, Salsman J, Conrad DM, Hoskin DW (2005). Bovine lactoferricin selectively induces apoptosis in human leukemia and carcinoma cell lines. Mol Cancer Ther.

[26] Yoo YC, Watanabe R, Koike Y, Mitobe M, Shimazaki K (1997). Apoptosis in human leukemic cells induced by lactoferricin, a bovine milk protein-derived peptide: involvement of reactive oxygen species. Biochem Biophys Res Commun.

[27] Yoo YC, Watanabe S, Watanabe R, Hata K, Shimazaki K (1997). Bovine lactoferrin and lactoferricin, a peptide derived from bovine lactoferrin, inhibit tumor metastasis in mice. Jpn J Cancer Res.

[28] Berge G, Eliassen LT, Camilio KA, Bartnes K, Sveinbjornsson B (2010). Therapeutic vaccination against a murine lymphoma by intratumoral injection of a cationic anticancer peptide. Cancer Immunol Immunother.

[29] Eliassen LT, Haug BE, Berge G, Rekdal O (2003). Enhanced antitumour activity of 15-residue bovine lactoferricin derivatives containing bulky aromatic amino acids and lipophilic N-terminal modifications. J Pept Sci.

[30] Yang N, Lejon T, Rekdal O (2003). Antitumour activity and specificity as a function of substitutions in the lipophilic sector of helical lactoferrin-derived peptide. J Pept Sci.

[31] Yang N, Stensen W, Svendsen JS, Rekdal O (2002). Enhanced antitumor activity and selectivity of lactoferrin-derived peptides. J Pept Res.

[32] Yang N, Strom MB, Mekonnen SM, Svendsen JS, Rekdal O (2004). The effects of shortening lactoferrin derived peptides against tumour cells, bacteria and normal human cells. J Pept Sci.

[33] Sen TZ, Jernigan RL, Garnier J, Kloczkowski A (2005). GOR V server for protein secondary structure prediction. Bioinformatics.

[34] Mosmann T (1983). Rapid colorimetric assay for cellular growth and survival: application to proliferation and cytotoxicity assays. J Immunol Methods.

[35] Livak KJ, Schmittgen TD (2001). Analysis of relative gene expression data using real-time quantitative PCR and the 2^−ΔΔ*C*T^ method. Methods.

[36] Zeya HI, Spitznagel JK (1963). Antibacterial and enzymic basic proteins from leukocyte lysosomes: separation and identification. Science.

[37] Zeya HI, Spitznagel JK (1966). Cationic proteins of polymorphonuclear leukocyte lysosomes I. Resolution of antibacterial and enzymatic activities. J Bacteriol.

[38] Pereira A, Kerkis A, Hayashi MA, Pereira AS, Silva FS (2011). Crotamine toxicity and efficacy in mouse models of melanoma. Expert Opin Investig Drugs.

[39] Overwijk WW, Restifo NP (2001). B16 as a mouse model for human melanoma. Current PROTOCOLS IN IMMUNOLOGY.

[40] Barasch A, Epstein JB, Foong WC, Clayman L (2004). Intralesional chemotherapy for head and neck carcinoma: a review of the literature. Oral Surg Oral Med Oral Pathol Oral Radiol Endod.

[41] Cornwell DG, Heikkila RE, Bar RS, Biagi GL (1968). Red blood cell lipids and the plasma membrane. J Am Oil Chem Soc.

[42] Britten CD, Rowinsky EK, Baker SD, Agarwala SS, Eckardt JR (1999). A phase I and pharmacokinetic study of temozolomide and cisplatin in patients with advanced solid malignancies. Clin Cancer Res.

[43] Lee SM, Margison GP, Thatcher N, O’Connor PJ, Cooper DP (1994). Formation and loss of O6-methyldeoxyguanosine in human leucocyte DNA following sequential DTIC and fotemustine chemotherapy. Br J Cancer.

[44] Lens MB, Eisen TG (2003). Systemic chemotherapy in the treatment of malignant melanoma. Expert Opin Pharmacother.

[45] Chan JK, Roth J, Oppenheim JJ, Tracey KJ, Vogl T (2012). Alarmins: awaiting a clinical response. J Clin Invest.

[46] Pisetsky DS (2014). The translocation of nuclear molecules during inflammation and cell death. Antioxid Redox Signal.

[47] Fiuza C, Bustin M, Talwar S, Tropea M, Gerstenberger E (2003). Inflammation-promoting activity of HMGB1 on human microvascular endothelial cells. Blood.

[48] Messmer D, Yang H, Telusma G, Knoll F, Li J (2004). High mobility group box protein 1: an endogenous signal for dendritic cell maturation and Th1 polarization. J Immunol.

[49] DeMarco RA, Fink MP, Lotze MT (2005). Monocytes promote natural killer cell interferon gamma production in response to the endogenous danger signal HMGB1. Mol Immunol.

[50] Dinarello CA (2009). Immunological and inflammatory functions of the interleukin-1 family. Annu Rev Immunol.

[51] Nakae S, Asano M, Horai R, Iwakura Y (2001). Interleukin-1β, but not interleukin-1α, is required for T-cell-dependent antibody production. Immunology.

[52] Bettelli E, Carrier Y, Gao W, Korn T, Strom TB (2006). Reciprocal developmental pathways for the generation of pathogenic effector T_H_17 and regulatory T cells. Nature.

[53] Chaves F, Teixeira CF, Gutierrez JM (2005). Role of TNF-α, IL-1β and IL-6 in the local tissue damage induced by *Bothrops asper* snake venom: an experimental assessment in mice. Toxicon.

[54] Biet F, Locht C, Kremer L (2002). Immunoregulatory functions of interleukin 18 and its role in defense against bacterial pathogens. J Mol Med (Berl).

[55] Hoshino T, Kawase Y, Okamoto M, Yokota K, Yoshino K (2001). Cutting edge: IL-18-transgenic mice: in vivo evidence of a broad role for IL-18 in modulating immune function. J Immunol.

[56] Nakanishi K, Yoshimoto T, Tsutsui H, Okamura H (2001). Interleukin-18 is a unique cytokine that stimulates both Th1 and Th2 responses depending on its cytokine milieu. Cytokine Growth Factor Rev.

[57] Okamura H, Tsutsui H, Komatsu T, Yutsudo M, Hakura A (1995). Cloning of a new cytokine that induces IFN-γ production by T-Cells. Nature.

[58] Shaw G, Kamen R (1986). A conserved AU sequence from the 3′ untranslated region of GM-CSF mRNA mediates selective mRNA degradation. Cell.

