# Supplementary material for: Complete regression and systemic protective immune responses obtained in B16 melanomas after treatment with LTX-315
Source: Cancer Immunol Immunother. 2014 Mar 28;63(6):601–13. doi: 10.1007/s00262-014-1540-0 (PMC4024132; doi:10.1007/s00262-014-1540-0)
Supplement: Supplementary file 1 — Supplementary material 1 (PDF 91 kb) [file 262_2014_1540_MOESM1_ESM.pdf]

Supplementary Table 1: Primers used for RT-qPCR

Gene symbol (mouse)	Name	Cat.No.	Amplicon length (bp)	Gene Bank Accession Number
IL1b	Interleukin 1beta	QT01048355	150	NM_008361
IL2	Interleukin 2	PPM02937C	111	NM_008366.3
IL4	Interleukin 4	QT00160678	104	NM_021283
IL6	Interleukin 6	QT00098875	128	NM_031168
IL10	Interleukin 10	PPM03017C	81	NM_010548.2
IL12b	Interleukin 12b	QT00153643	97	NM_008352
IL18	Interleukin 18	QT00171129	149	NM_008360
Tnf	Tumor necrosis factor	QT00104006	112	NM_013693
Ifng	Interferon gamma	PPM03121A	95	NM_008337.3
Tgfbrap	Transforming growth factor, beta receptor associated protein 1	QT01543241	102	NM_001013025
Actb	Actin, beta	QT01136772	77	NM_007393
Gapdh	Glyceraldehyde-3-phosphate dehydrogenase	QT01658692	144	NM_008084

Cancer Immunology, Immunotherapy (submitted in 2014) – Ketil André Camilio et al.
